# Collagen-producing eye cell atlas reveals distinct fibroblast fates in early injury vs. fibrotic subretinal disease

**DOI:** 10.1073/pnas.2519056123

**Published:** 2026-06-26

**Authors:** Ema Ozaki, Said Aktas, Kelly Mulfaul, Kiva Brennan, Christophe Roubeix, Sarah Palko, Katie Robb, Tai-Hsien Ou Yang, Marie-Claire Schanne-Klein, Anna Toidze, Avril Watson, Mark Cahill, Peter D. Westenskow, Derrick Feenstra, Sarah L. Doyle

**Affiliations:** ^a^ Department of Clinical Medicine, School of Medicine, Trinity College Dublin, Dublin 2, Ireland; ^b^ Trinity College Institute of Neuroscience, Trinity College Dublin, Dublin 2, Ireland; ^c^Roche Pharma Research and Early Development, Roche Innovation Center Basel, F. Hoffmann-La Roche Ltd., 4058 Basel, Switzerland; ^d^Pharma Research and Early Development, Roche Innovation Center Zurich, Roche Glycart AG., Schlieren 8952, Switzerland; ^e^ Department of Neuroscience and Pharmacology, University of Iowa, Iowa City, IA 52242-1109; ^f^ Institute for Vision Research, University of Iowa, Iowa City, IA 52242-1091; ^g^Progressive Vision Research, Dublin 18, Ireland; ^h^ The Royal Victoria Eye and Ear Hospital, Dublin 2, Ireland; ^i^Roche Pharma Research and Early Development, Data and Analytics, Roche Translational & Clinical Research Center, F. Hoffmann-La Roche Ltd., Little Falls, NJ 07424; ^j^ Laboratoire d’Optique et Biosciences, Ecole Polytechnique, CNRS, INSERM, Institut Polytechnique de Paris, Palaiseau 91120, France; ^k^ Department of Biosystems Science and Engineering, ETH Zurich, Basel 4056, Switzerland

**Keywords:** collagen-atlas, scRNA-sequencing, subretinal fibrosis, fibroblast, neovascular age-related macular degeneration

## Abstract

Fibrosis is the end-stage of a maladaptive process that occurs when the body’s normal wound-healing strategy becomes dysregulated. Subretinal fibrosis is the end stage of neovascular age-related macular degeneration (nAMD), the most common cause of central vision loss in people over the age of 50. The cellular sources of excess extracellular matrix (ECM) contributing to subretinal fibrosis are unknown, as is the heterogeneity of cells involved in the fibrotic process. Here we identify cells contributing to subretinal fibrosis by using *Col1a1*-YFP reporter mice to noninvasively image collagen production in real-time in vivo in two disease models, 1) a resolving retinal injury model and 2) a fibrotic model of subretinal disease. We create a collagen-producing eye cell atlas for subretinal injury and demonstrate subretinal fibroblast heterogeneity in healthy, resolving, and fibrotic tissue. We identify distinct molecular characteristics of general repair/resolving fibroblast populations versus pathogenic pro-fibrotic collagen-producing fibroblasts. Integration of this collagen-producing eye cell atlas with a published collagen-producing lung cell atlas shows conserved pro-fibrotic fibroblasts in both organs, yet also uncovers tissue-specific fibroblast populations unique to subretinal fibrosis. A *Fap^+^Fgl2^+^* fibroblast population significantly expands in subretinal fibrosis that expresses the highest levels of collagens and distinctively expresses ECM components *Periostin*, *Col15a1* and *Col6a5*. Immunolabeling of mouse and human-donor eye tissue support the fibroblastic expression and perivascular location of periostin as clearly distinguishing between bona fide fibrosis and early disease in nAMD. Our collagen-producing eye cell atlas is a valuable resource for studying distinct fibroblast subsets in homeostasis, early injury, and fibrosis.

## Significance

The cellular sources of excess extracellular matrix (ECM) contributing to subretinal fibrosis in Age-related macular degeneration are unknown. Our comparative-injury model method advances the field demonstrating that choroidal fibroblasts are a dominant source of ECM in subretinal fibrosis, while identifying differing fibroblast fates in resolving versus fibrotic disease. scRNA-seq and cross organ integration studies identify molecular characteristics of universal general repair/resolving fibroblasts and pathogenic pro-fibrotic collagen-producing fibroblasts. Specialized fibroblasts with distinct nonoverlapping immunoregulatory or fibrillogenic characteristics emerge with injury but are common to both resolving and fibrotic disease. However, we also identify a retinal tissue-specific *Fap^hi^Fgl2^+^Postn*^+^ fibroblast population expressing a unique repertoire of ECM components that specifically expands in subretinal fibrosis which is validated in human donor eye tissue.

Fibrosis is the end stage of a maladaptive process that occurs when the body’s normal wound healing strategy becomes dysregulated. Effective therapies to prevent or resolve fibrosis are currently lacking, and fibrotic diseases of the skin, lung, liver, and kidney contribute to 45% of all-cause mortality (1). Perturbations in collagen homeostasis is an important contributing factor in fibrotic disease and subdivisions within fibroblasts that segregate functionally to overproduce excessive extracellular matrix (ECM) have been identified in a variety of soft tissue fibrotic diseases. Deciphering fibroblast heterogeneity between homeostasis and disease states across multiple organs is generating insights into pathogenic fibroblast phenotypes providing a greater understanding of the roles of pan-tissue and tissue-specific fibroblasts in fibrotic disease.

Fibrotic disease can also affect our sight. Retinal neovascular diseases such as neovascular age-related macular degeneration (nAMD) are characterized by the formation of pathological new blood vessels that leak plasma into the retina leading to vision loss. AMD alone is the leading cause of irreversible central blindness affecting around 200 million people globally (2), and while current therapies lead to an improvement in visual acuity for many patients with nAMD, longitudinal studies indicate that in up to ~50% of nAMD patients there is a switch from a pro-angiogenic to a pro-fibrotic state within 2 y of first treatment (3). In this phase, choroidal blood vessels undergo progression to fibrovascular tissue and the subsequent excessive scar formation limits visual gains.

The mechanisms underlying development of subretinal fibrosis secondary to nAMD are unknown, however, excessive ECM deposition of collagen appears to be a key event. The cells responsible for excess collagen production in subretinal fibrosis are not well characterized, mainly because fibroblasts are not a resident cell in the healthy retina, and there is a lack of information on mesenchymal cell subtypes in tissue subjacent to the retina. One study by Luo et al. using *Col1a1*-GFP reporter mice in conjunction with immunohistochemistry has suggested PDGFRb+ pericytes are significant contributors to subretinal fibrosis (4). However, as multiple stromal cell types express PDGFRb the main cellular contributors of excess ECM in subretinal fibrosis remains an open question. Furthermore, the majority of studies have focused on epithelium-to-mesenchymal transition of the retinal pigment epithelial (RPE) cells, and reports on fibroblasts in subretinal disease are scarce.

In order to develop therapeutic strategies to prevent subretinal fibrosis in patients or be in a position to repurpose emerging anti-fibrotic therapeutics for subretinal fibrosis, we need a better understanding of the cells responsible for excess collagen production, remodeling, and scar formation in the retina. Integrated scRNA sequencing data imply the fibroblast lineage is compartmentalized into three major subtypes, universal and tissue-specialized ‘steady-state’ subsets as well as ‘activated’ subsets (5). Identifying pro-fibrotic fibroblast phenotypes for therapeutic targeting without interfering with other fibroblast subsets involved with repair or tissue specialization would represent a great clinical benefit (6). Using transgenic *Col1a1*-YFP mice, this study focused on identifying the cells responsible for the overproduction of collagen and other ECM proteins by performing single cell RNA sequencing (scRNA seq) of all *Col1a1*-producing cells. To further our understanding of mechanisms promoting maladaptive pro-fibrotic biological processes in place of self-limiting wound-healing processes, we modified the classic model of laser-induced choroidal neovascularization (LCNV) and established a timeline for identifying collagen-producing cells in resolving neovascular disease versus fibrotic disease. We find that the cells responsible for the production of *Col1a1* in subretinal fibrosis are fibroblasts located in the RPE/choroid compartment. We identify highly specialized fibrillogenic and immunoregulatory fibroblast clusters that represent repair fibroblast clusters. We identify two collagen-producing pathogenic fibroblast clusters that are overrepresented in fibrotic tissue, representing pro-fibrotic fibroblasts. One of these subsets is enriched for *Cthrc1* and *Spp1* and therefore bears the signature of a general universal pathogenic fibroblast observed in other nonocular fibrotic disease (7). The other subset is enriched for *Postn* and other collagens, and we identify periostin as a specific biomarker, distinguishing fibrotic tissue from tissue undergoing active remodeling in both mouse and nAMD human tissue sections. Integration of our murine dataset with fibrotic tissue from nonocular soft tissue disease finds a commonality in fibroblasts emphasizing the conservation of mechanisms of fibrosis across tissues and organs. Our data pertaining to fibroblast heterogeneity in resolving disease and subretinal fibrosis should therefore have broad applicability for a variety of other soft tissue fibrotic diseases.

## Results

### Col1a1-Producing Cells Are Sustained at the Neovascular Lesion in the Subretinal Space After Repeated Laser Injury.

**Fig. 1.**
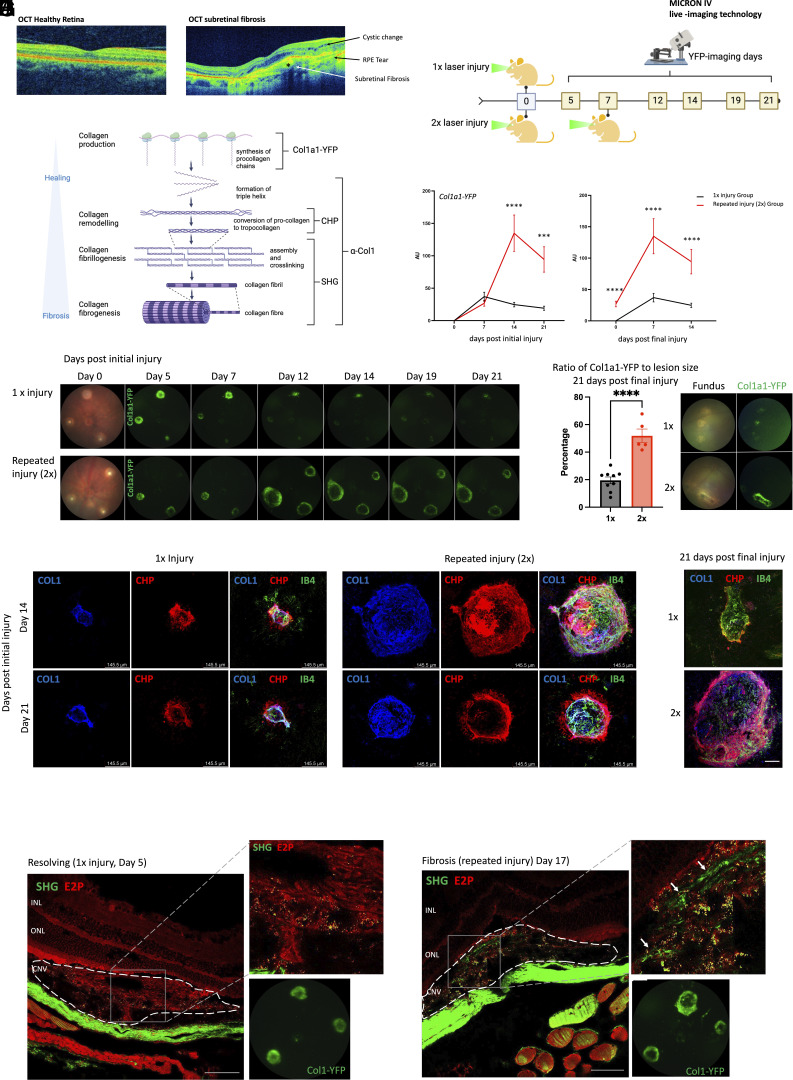
Prolonged presence of *Col1a1*-producing cells leads to stable collagen fibril formation resulting in subretinal scar tissue. (*A*) OCT scan of retina from a healthy patient and AMD patient with subretinal fibrosis; asterisk marks fibrotic region. (*B*) Schematic of collagen synthesis stages and assessment tools. (*C*) Timeline of laser injury and YFP imaging. (*D* and *E*) Quantification and in vivo fundus and YFP imaging of *Col1a1*-YFP mice over 21 d post LCNV [top row 1× LCNV on Day 0, bottom row repeated (2×) LCNV on Day 0 and Day 7] using the Micron IV platform. Quantification of YFP fluorescence in *Left* panel depicts days post initial injury, *Right* panel depicts days post final injury (n = 17, ***P ≤ 0.001, ****P ≤ 0.0001). (*F*) Ratio of *Col1a1*-YFP to lesion size 21 d post final injury, top row 1× LCNV, bottom row 2× LCNV (n > 5 ≤ 0.001, ****P ≤ 0.0001). (*G*) RPE flatmount from WT mice 14 and 21 d post initial laser injury, 1× LCNV (*Left*) and repeated 2× LCNV (*Right*) stained with COL1, CHP, and IB4. (*H*) RPE flatmount from WT mice 21 d post final laser injury for 1× LCNV (*Top*) and repeated 2× LCNV (*Bottom*) stained with COL1, CHP, and IB4. (*I*) Retinal cryosection from *Col1a1*-YFP mice 5 d post 1× LCNV (*Left*) and (*J*) 17 d post repeated 2× LCNV (*Right*) imaged by SHG and E2P microscopy (merged) to detect fibrillar collagens, with a magnified image shown on the *Top Right* and a representative in vivo YFP image from LCNV in *Col1a1*-YFP mice shown below. White arrows indicate regions of collagen fibrils.

In approximately 50% of patients with wet AMD, the disease will progress to a fibrotic stage within 2 y of treatment (3). OCT images are shown of a healthy retina (Fig. 1 *A*, *Left* panel) and an eye with subretinal fibrosis in the macular region (Fig. 1 *A*, *Right* panel), with the center of the area of scarring depicted by an asterisk. Collagen biogenesis is important for wound healing processes but excess accumulation of mature fibrillar collagen is the hallmark of fibrotic disease (Fig. 1*B*). To study the cells involved in excess collagen accumulation in subretinal fibrosis we established parameters for self-limiting wound repair and fibrotic disease in the retina. We employed a time course of noninvasive in vivo YFP-imaging after 1× laser injury out to 21 d post initial injury in *Col1a1*-YFP reporter mice. We compared this with a second cohort of *Col1a1*-YFP reporter mice given a second laser injury 1 wk post initial administration, reasoning that fibrosis occurs in response to chronic injury (2× laser injury) (8) (Fig. 1*C*). Using Micron IV fluorescent live imaging technology, we found *Col1a1*-producing cells appeared strongly at day 5 in response to 1× laser injury, but the abundance of collagen-production waned from 7 d post injury (Fig. 1 *D*, *Left* panel and Fig. 1 *E* representative images, *Top* panel). In contrast, when a second laser injury was administered, the abundance of *Col1a1*-producing cells increased, and their presence was prolonged and relatively stable from day 12 out to day 21 post initial injury (Fig. 1 *D*, *Left* panel and Fig. 1 *E* representative images, *Bottom* panel). To ascertain if the difference in persistence of *Col1a1*-producing cells between the two models was simply due to the differences in time passed since the final laser injury, we quantified the *Col1a1*-producing cells at equivalent time points post final laser injury. Comparisons of collagen-production post *last* injury clearly demonstrated that collagen-production was significantly increased in the 2× compared with the 1× model at equivalent timepoints following injury (Fig. 1 *D*, *Right* panel). Extending out the timeframe for comparison of *Col1a1*-producing cells between models to 21 d post final injury (Fig. 1*F*) further supports a conclusion that the two injury models represent distinct outcomes; injury resolution vs. persistence.

### Sustained Presence of Col1a1-Producing Cells Coincides with Stable Collagen Fibril Formation.

To assess if prolonged presence of *Col1a1*-producing cells in the 2× model corresponded with an increase in COL1 deposition and modeling, we used an antibody targeting COL1, in addition to collagen hybridizing peptides (CHP), on RPE flatmounts prepared at day 14 and day 21 post initial laser injury from mice administered 1× or 2× injury. As expected the higher abundance of *Col1a1*-producing cells in the 2× injury model corresponded with a larger volume of COL1 deposition at both day 14 and day 21 compared with the equivalent days in the 1× injury model (Fig. 1*G*, blue staining). Interestingly, the pattern of CHP staining was notably different within the 2× injury model between day 14 and day 21 post initial injury, with CHP staining visible only at the outer edges of the neovascular lesion by day 21, compared with full coverage of CHP across the lesion at day 14 (Fig. 1*G*, red staining). While the COL1 antibody detects all forms of this protein, CHPs specifically identify collagens undergoing active modeling as they combine with open collagen fibers (Fig. 1*B*). Therefore, our data imply the process of collagen remodeling associated with tissue repair and healing is ongoing at day 14, however, by day 21 we observe a ‘cold’ COL1^+^CHP^−^ fibrotic scar in the central area of the lesion. Labeling for CHP at the later, equivalent timepoint of 21 d post final laser injury in both models demonstrated a marked difference in CHP presence, supporting this interpretation, with remodeling persisting in the 2× model and practically absent from the 1× model at this later timepoint (Fig. 1*H*). During scarring, collagen microfibrils self-assemble into higher-order structures to form fibrils, and then fibers, which are stabilized through covalent cross-linking (Fig. 1*B*). To confirm collagen-fibrillogenesis was occurring in the 2× model, we employed second harmonic generation (SHG) microscopy to identify the presence of fibrillar collagen (9, 10). Two-photon excited fluorescence signals detected from endogenous cellular fluorophores enabled visualization of tissue morphology (11). Despite the presence of *Col1a1*-YFP producing cells and COL1 deposition at day 5 in the 1× injury model, we did not observe fibrillar collagen in the CNV lesion at this timepoint (Fig. 1*I*). In contrast, we found evidence of fibrillar collagen in the CNV lesion at day 17 following 2× injury (Fig. 1*J*), indicating that fibrillogenesis and mechanisms resulting in fibrosis are active under these parameters.

### scRNA Seq Analysis Reveals Expansion of Choroidal Fibroblast Heterogeneity in Subretinal Neovascular Lesions.

**Fig. 2.**
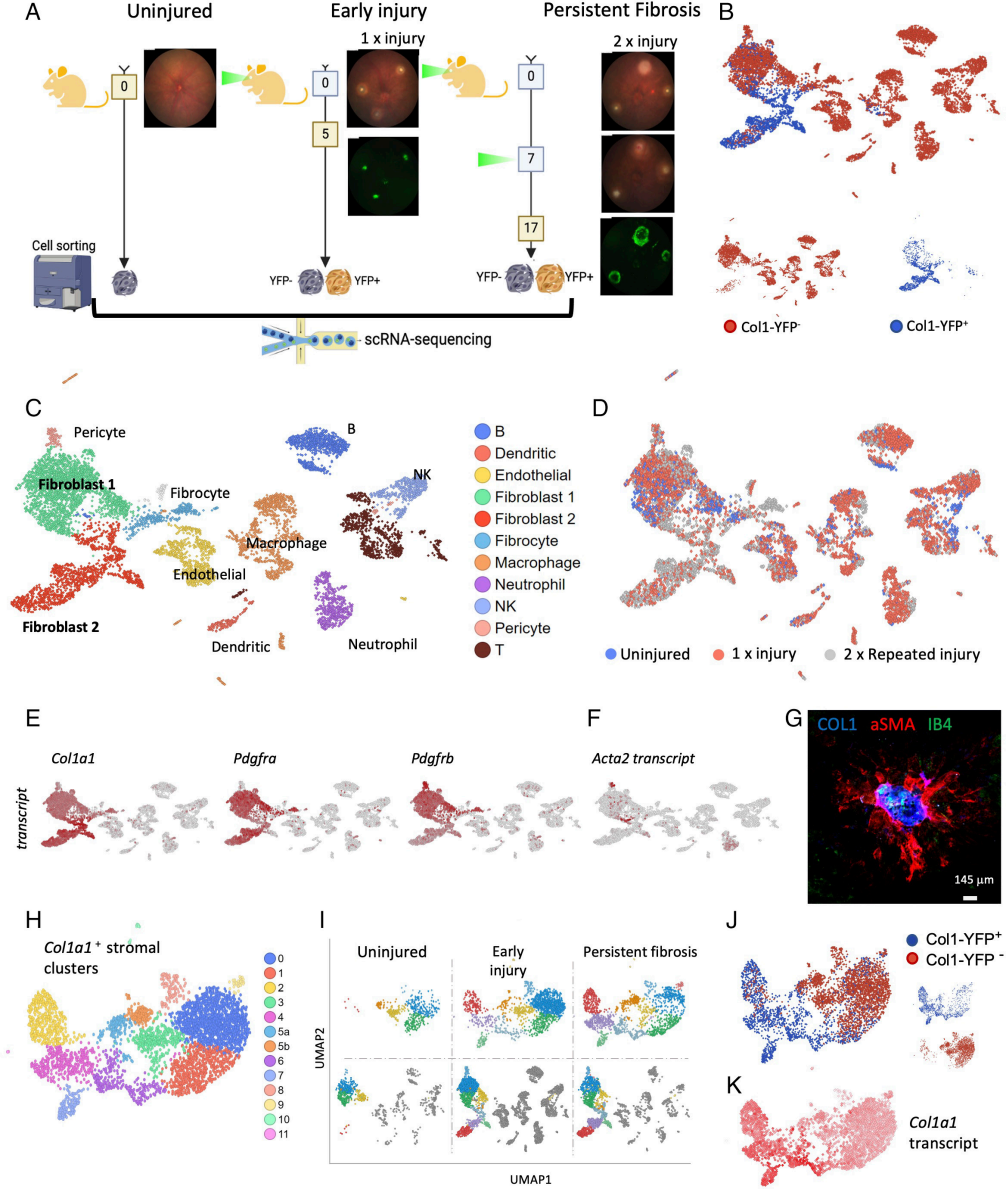
Single cell RNA seq reveals choroidal fibroblasts are the main Col1a1-producing cells in subretinal neovascular lesions. (*A*) Workflow for injury induction, imaging, YFP+ sorting, and 10× scRNA-seq. (*B*–*D*) Uniform manifold approximation and projection (UMAP) plot of all cells categorized by (*B*) YFP status, (*C*) cell type, and (*D*) disease state (uninjured, early injury, fibrosis). (*E* and *F*) UMAPs showing expression of *Col1a1, Pdgfra, Pdgfrb,* and *Acta2*. (*G*) RPE flatmount from WT mice 14 d post LCNV stained with αSMA, Isolectin GS-Ib_4_ (IB4), and Collagen Type I (COL1). (*H*) Reclustered Col1a1+ cells from the current subretinal-RPE/choroid study produced 12 new clusters, UMAP. (*I*) UMAP of new clusters separated into uninjured, early injury and fibrosis samples (top) mapped onto UMAP of all cells (bottom). (*J*) UMAP of reclustered Col1a1+ cells colour-coded into YFP status. (*K*) UMAP of Col1a1 expression.

Potential cellular sources of collagen and excess ECM at the neovascular lesion include choroidal fibroblasts, choroidal endothelial cells, circulating fibrocytes, RPE or neural retinal cells such as pericytes, and activated glial cells. To identify high *Col1a1*-producing cells in subretinal fibrosis we designed a method to utilize the *Col1a1*-YFP reporter mouse and single cell RNA-sequencing in conjunction with our parameters for laser injury to compare between early response to injury and fibrotic disease. Using fundus photography and fluorescent live imaging, we confirmed *Col1a1*-producing cells were present at day 5 in 1× model, and at day 17 in 2× model (Fig. 2*A*, representative images). We compared the percentage of *Col1a1*-producing cells between retinal tissue and RPE/choroid tissue by flow cytometry and found practically no *Col1a1*-producing cells in the neural retinal isolate in either injury-model, whereas *Col1a1*-producing cells were found in the RPE/choroid tissue (*SI* *Appendix,* Fig. S1*A*). For this reason we performed scRNA-seq using 10X Genomics on YFP^+^ and YFP^−^ cells sorted from the RPE/choroid tissue of *Col1a1*-YFP mice (Fig. 2*A*). When we compared *Col1a1*-YFP^+^ cells (blue) with *Col1a1*-YFP^−^ cells (red), we found the majority of *Col1a1*-producing cells clustered together (Fig. 2*B*). Cell cluster annotation demonstrates a wide range of cells present in the RPE/choroid tissue in all conditions with the exception of a large distinct fibroblast cluster (Fibroblast 2) that is practically absent in healthy tissue (Fig. 2 *C* and *D* and *SI* *Appendix,* Fig. S1 *B* and *C*). Fibroblast clusters were clearly identifiable by their high expression of *Pdgfra*/*Col1a1/Col1a2* and distinguishable from pericytes identifiable as *Pdgfra*^−^/*Pdgfrb*^+^/*Rgs5^+^*/*Cspg4^+^*/*Notch3^+^* and fibrocytes identified as *Pdgfra*^+^/*Ptprc*^+^ (Fig. 2*E* and *SI* *Appendix,* Fig. S1*D*).

### scRNA Seq Analysis Reveals Distinct Cluster of Choroidal Fibroblasts Are the Main Col1a1-Producing Cells in Subretinal Neovascular Lesions.

Transcriptomic expression of *Col1a1* corresponds well with *Col1a1*-YFP expression (Fig. 2 *B* and *E*). Percentage cell composition for each of the five samples demonstrates the appearance of fibrocytes in fibrosis over early injury (*SI* *Appendix*, Fig. S1), however despite the presence of *Col1a1* mRNA transcript in fibrocytes, the majority of fibrocytes are *Col1a1*-YFP^−^ indicating low translation of COL1 protein in these cells. We observe a greater percentage of *Col1a1*-producing macrophages in early injury compared to fibrosis, however, these represent a small population of *Col1a1*-producing cells, ~5%. Overall, the majority of cells actively translating *Col1a1* into protein are choroidal fibroblasts. Within this cell grouping there is a distinct division in collagen expression, with the majority YFP^+^ collagen-producers in the cluster that appears in response to injury (fibroblast 2), which we now term disease-associated fibroblasts (DAFs) (Fig. 2 *B*–*D*). Much of the literature on subretinal fibrosis is focused on αSMA^+^ mesenchymal cells, however, there was a lack of *acta2*, the gene that encodes αSMA, in the subretinal DAFs (Fig. 2*F*). We confirmed this in tissue flatmounts (Fig. 2*G*) where we observed abundant αSMA labeling in the area adjacent to the nascent isolectin GS-IB_4_ (IB4)^+^ vessels. However, while there are some distinct areas of colocalization observed between αSMA^+^ cells and COL1 (open arrow), in the main COL1 labeling did not colocalize with αSMA indicating that the primary cell types responsible for COL1 deposition are αSMA^lo^ or αSMA^−^.

### Integration of Stromal Cell scRNA-seq Datasets Reveals Similarities and Tissue-Specific Distinctions in DAFs from Subretinal and Nonocular Fibrotic Disease.

Recent studies have identified universal and tissue-specific, steady-state, and activated fibroblast subsets in stromal tissue from across a variety of organs. We integrated our subretinal dataset with a lung dataset that used a similar study design to ours, incorporating *Col1a1*-reporter mice in their scRNA seq analysis to identify DAFs (7). Stromal cells from both tissues segregated into eighteen clusters (*SI Appendix*, Fig. S2). Analysis of annotation- and study-identifying UMAPs revealed tissue origin is a strong driver for cell clustering. However, homeostatic or “steady state” fibroblasts (clusters 2,8,12), comprised of roughly equal proportions of fibroblasts from both subretinal and lung uninjured states, had representation from both tissues indicating these are universal steady-state fibroblasts. DAFs also had had representation from both tissues. In fact, when we looked specifically at lung-fibrosis DAF marker *Cthrc1* we found it most highly expressed in the area on the UMAP where lung and mouse DAFs overlap *SI Appendix*, Fig. S2, indicating that our dataset contains both tissue-specific and universal DAF phenotypes. A dotplot showing the mean gene expression and fraction of expressing cells in the clusters revealed that, in general, the fraction of DAFs expressing known markers of fibrosis were similar across tissues, however the mean expression of the fibrotic markers was higher in cluster 9 that contains cells from both tissues.

### Collagen-Producing Cell Atlas Reveals Col1a1-YFP^−^ and Col1a1-YFP^+^ Fibroblasts Segregate with Tissue Injury.

Confident that we had isolated and identified distinct homeostatic steady-state fibroblasts, and activated DAFs we focused in on the *Col1a1^+^* stromal cells, and found they robustly clustered into 12 distinct subgroups (Fig. 2*H* and *SI* *Appendix,* 3). Three fibroblast clusters were strongly represented in healthy tissue (clusters 0, 1, 3) (Fig. 2 *H* and *I*), and mapped extremely well with *Col1a1*-YFP^−^ cells (Fig. 2*J*). Four more fibroblast clusters appeared in response to injury (clusters 2, 4, 6, 7) (Fig. 2 *H* and *I*). These four injury or disease-associated clusters mapped with higher *Col1a1* mRNA and *Col1a1*-YFP^+^ expression (Fig. 2 *J* and *K*). Viewing these clusters across the tissue states [uninjured, (1×) early injury and (2×) fibrosis], we observed a shift in the majority cluster grouping present in fibrosis compared to resolving injury. In early injury the majority of clusters are those that are also present in healthy tissue (i.e., clusters 0, 1, 3). However, in fibrosis while these ‘homeostatic fibroblast’ (HF) clusters remain present, the balance has tipped in favor of a majority of DAF clusters (i.e., clusters 2, 4, 6, 7), with the appearance of a further fibrosis-associated cluster (cluster 9), which are interferon-responsive but *Col1a1*-YFP^−^ fibroblasts (Fig. 2*I* and *SI* *Appendix,* Fig. S3). Further analysis of the fibrocyte cluster 5 identified a *Nrp1*^-^homeostatic fibroblast cluster 5a and a true fibrocyte cluster 5b only found in fibrosis, but *Col1a1*-YFP^−^ (Fig. 2*I*).

### Multiple ECM Producing and Degrading Pathways Coexist in Col1a1-YFP^+^ DAFs.

**Fig. 3.**
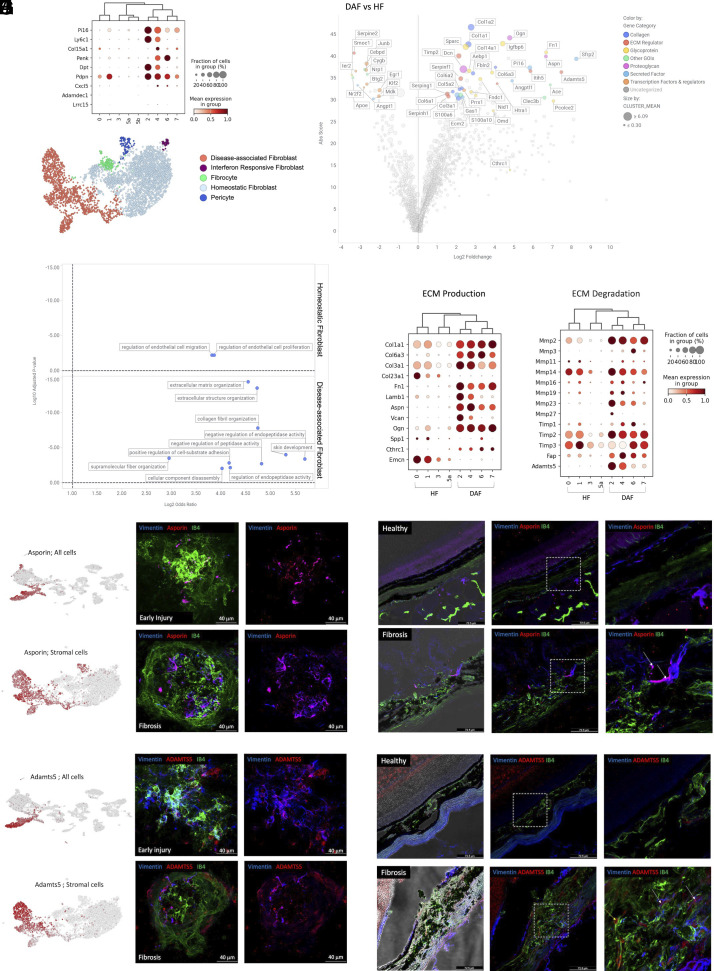
ECM generation and degradation pathways coexist in DAFs in subretinal fibrosis. (*A*) Dot plot displaying dominant genes described for fibroblasts in perturbed tissue. UMAP of reclustered stromal cells color-coded by cell subtype. (*B*) GSEA pathways enriched in HFs and DAFs, (*C*) Volcano plot showing highest DEGs in DAFs compared to HFs. (*D*) Dot plot of genes involved in ECM generation and ECM degradation in the HF and DAF clusters. (*E* and *F*) UMAP of Aspn and Adamts5 in all cells and stromal cells. (*G*) RPE flatmounts from WT mice 5 d (early injury, 1×) and 17 d (fibrosis, 2×) post LCNV stained with vimentin, IB4, and Asporin. (*H*) Retinal cryosections from uninjured (healthy) and 17 d (fibrosis, 2×) post LCNV WT mice stained with vimentin, IB4, and Asporin, magnified images shown on the right. (*I*) RPE flatmounts from WT mice 5 d (early injury, 1x) and 17 d (fibrosis, 2x) post LCNV stained with vimentin, IB4, and ADAMTS5. (*J*) Retinal cryosections from uninjured (healthy) and 17 d (fibrosis, 2x) post LCNV WT mice stained with vimentin, IB4, and ADAMTS5, with magnified images shown on the right. White arrows depict areas of costaining.

Despite each DAF cluster having clearly distinctive, segregating gene expression profiles it is notable that one of the top DEGs found almost exclusively in all DAF clusters when compared with HF’s is *Pi16*, which identifies universal activated fibroblasts (12). In fact when we analyzed a panel of genes, identified in a large scale multitissue study on fibroblast phenotype enriched in ‘perturbed-tissue’ (5) we found expression of most genes in a minimum of two or more DAF clusters, with minimal to no expression in HF’s (Fig. 3*A*). This further confirmed our own ‘DAF’ annotation of these clusters for subretinal disease and also indicated our data was rich enough to parse out additional phenotypic detail. Initially however, pathway analysis of DEGs between clusters did not highlight enriched pathways, returning similar broad terms for all. Therefore, we grouped the HF clusters (0,1,3,5a) and DAF clusters (2,4,6,7) and examined the greatest differences between them across all models. Pathway analysis using gene set enrichment analysis (GSEA) highlights that while HFs actively regulate endothelial cell migration and proliferation, DAFs have upregulated pathways involved in ECM organization and collagen fibril formation, alongside regulating endopeptidase activity (Fig. 3*B*). We found that in addition to *Col1a1*, many other collagens (blue), glycoproteins (yellow), proteoglycans (pink) and proteases (red) have enhanced gene expression in DAFs compared with HFs (Fig. 3*C*, volcano plot). In fact a multiplicity of ECM producing and degrading pathways coexist in parallel in DAFs, with a majority expressed by all four clusters, and a minority expressed by only one or two clusters (Fig. 3*D*). A number of genes were highly polarized in expression, ECM producing genes in DAFs that are absent in HFs include *Fn1*, *Lamb1*, *Aspn*, *Ogn*, *Cthrc1,* and *Col6a3*, a fibrillar collagen similar to *Col1a1* that also produces the active fragment endotrophin. ECM degrading genes in DAFs that are absent in HFs include *Mmp3*, *Mmp23,* and *Adamts5*. In contrast, HFs express *Col23a1*, a relatively uncharacterized transmembrane collagen involved in cell adhesion (13), and *Emcn* a mucin-like sialoglycoprotein that inhibits interaction between cells and the ECM (14), both of which are absent in DAFs.

*Adamts5* and *Aspn* were two of the most highly DEGs in DAFs (Fig. 3*C*), with *Aspn* relatively well expressed across all DAF clusters (Fig. 3 *D*, *Left* panel) while *Adamts5* was most highly expressed in clusters 2 and 4 (Fig. 3 *D*, *Right* panel); both were practically absent from all other clusters (Fig. 3 *E* and *F*). Using vimentin to identify all fibroblasts, and asporin to identify DAFs, we verified the presence of DAFs in flatmounts of LCNV lesions in both early injury and fibrosis through vimentin/asporin double-positive labeling (Fig. 3*G*). Immunolabeling of tissue cryosections revealed comparatively little asporin in vimentin^+^ cells in healthy choroid or in the choroid subjacent to the RPE, whereas we observed strong vimentin/asporin double positive labeling across the lesion site in early injury and in the fibrosis model (Fig. 3*H*). These IHC data verified the scRNA expression data and the presence of DAFs at the LCNV lesion.

Together, Clusters 2 and 4 represent the largest proportion of DAFs in the fibrosis model (Fig. 2*I*) and also appear to express the widest variety of ECM producing and degrading genes across all fibroblast clusters (Fig. 3*D*). *Adamts5* stands out as a gene that appears to be relatively specific to these two clusters (Fig. 3*F*). To determine if we could observe the presence of these fibroblast clusters by IHC we examined tissue flatmounts (Fig. 3*I*) and cryosections of RPE/choroid (Fig. 3*J*), and confirmed the presence of vimentin/ADAMTS5 double-positive cells in early injury and fibrotic lesions but not in healthy RPE/choroid (Fig. 3*J*, white arrows). As expected we also observed vimentin^+^ ADAMTS5^-^ fibroblasts at the lesion site (Fig. 3*J*, yellow arrows). From these data we can extrapolate parallel expression of these matricellular proteins with opposing function in subretinal DAFs.

### Distinct Immunoregulatory and Fibrillogenic DAF States Predominate in Early Injury During Repair.

**Fig. 4.**
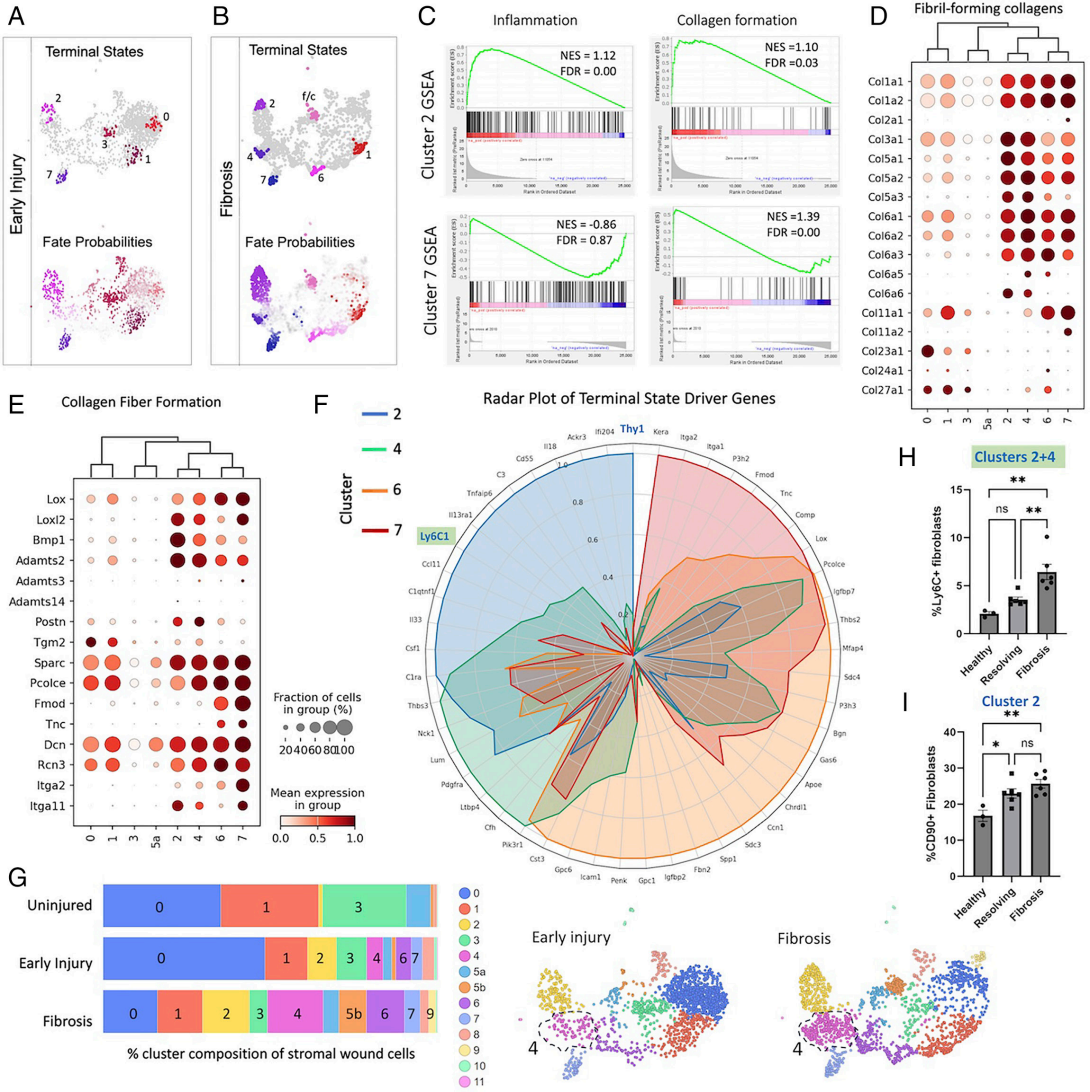
Distinct immunoregulatory and fibrillogenic states predominate early in subretinal injury, with expansion of poly-functional pathogenic fibroblast states in subretinal fibrosis. (*A* and *B*) Terminal state and fate probabilities UMAPs in (*A*) early injury and (*B*) fibrosis samples in the stromal cell clusters. (*C*) GSEA pathway analysis of cluster 2 and cluster 7, generated using the GSEA Preranked software. Normalized expression score and false discovery rate (FDR) are indicated on graphs. (*D* and *E*) Dot plot of genes involved in fibril-forming and collagen fiber formation in HF and DAF clusters. (*F*) Radial plot displaying expression levels of genes driving terminal states in DAF clusters. (*G*) Bar chart displaying percentage of each stromal cell cluster in healthy, early injury, and fibrosis tissue with UMAPs of stromal clusters in early injury and fibrosis shown alongside. (*H* and *I*) Flow cytometry analysis showing percentage of (*H*) Ly6C+ fibroblasts and (*I*) CD90+ (THY1) fibroblasts in RPE/choroid samples from uninjured mice and in resolving and fibrotic models.

It is noteworthy that there are practically no visible vimentin^+^ asporin^−^ cells at the LCNV lesion, suggesting that the HFs present in the tissue samples used for scRNA seq analysis are located in tissue at a distance from the LCNV and are not located at the site of injury itself with any abundance. With that in mind we wished to determine if there is a difference in the fate of DAF populations in early injury compared to a fibrotic tissue environment. We carried out terminal state and fate probability mapping of all stromal cells in early injury and fibrosis using the CellRank2 method (Fig. 4 *A* and *B*). In fibrosis, cells from each of the four DAF clusters were predicted to belong to terminal states, with the majority fated to cluster 2 (*Pi16^hi^ Thy1^hi^*) and cluster 7 (*Kera^hi^ Fmod^hi^*) phenotypes. Interestingly, these two clusters were identified as the only terminal DAF states in early injury. We utilized GSEA to delve deeper into potential specific roles of the *Pi16^hi^ Thy1^h^*^i^ cluster (cluster 2) and the *Kera^hi^ Fmod^hi^* cluster (cluster 7) using the list of driver genes revealed by CellRank2 (Fig. 4*C* and *SI* *Appendix,* Fig. S4*A*). We found strong phenotypic signatures that were highly polarized between these clusters, indicating that cluster 2 represents immunoregulatory fibroblasts, and cluster 7, that exhibited a negative correlation with immune-related genes, instead exhibited high enrichment for genes involved in collagen-fiber formation (Fig. 4*C*). Dot plots of collagen-crosslinking and collagen-fiber forming genes confirmed a strong fibrillogenic signature for cluster 7 (Fig. 4 *D* and *E*) demonstrating that cluster 7 has the highest expression not only of *Col1a1* but also of key collagen-crosslinking enzymes *Lox* and *Loxl2*. *Loxl2* is completely absent in HFs and indeed cross sections of healthy tissue are devoid of LOXL2 labeling. Expression of LOXL2, however, is not exclusive to fibrosis as we observe LOXL2 in tissue flatmounts of both early injury and fibrosis, with some vimentin^+^ cells expressing high LOXL2 compared to others likely marking out the *Kera^hi^ Fmod^hi^* cluster 7 fibroblasts (*SI* *Appendix,* Fig. S4 *B*–*D*). A radar plot of the top driver genes identified for the four terminal DAF states in fibrosis clearly demonstrates these distinct immunoregulatory and fibrillogenic signatures, are virtually mutually exclusive for clusters 2 and 7, respectively (Fig. 4*F*) which appear to have committed to specialize in immune or fibrillogenic function.

### Identification of Poly Functional Fibrogenic Fibroblast States that Expand in Subretinal Fibrosis.

Together *Pi16^hi^ Thy1^+^* (cluster 2) and *Kera^hi^ Fmod^hi^* (cluster 7) clusters represent a large proportion of terminally fated fibroblasts in subretinal fibrosis, however, these fibroblast clusters are also found in substantial numbers in early injury (Fig. 4*A*). In fact, cluster composition analysis indicates that these populations are not substantially different between early injury and fibrosis (Fig. 4*G*) meaning they likely represent the main fibroblast phenotypes involved in normal repair and resolution processes. On the other hand, cluster composition analysis of stromal wound cells across the three tissue states indicates that fibroblast clusters 4 (*Fap*^+^*Fgl2*^+^) and 6 (*Cthrc1^hi^Mmp3^+^Spp1^+^*) are not only increased in number in fibrosis compared to early injury (Fig. 4*G*), but also represent terminally differentiated fibroblast states only in fibrosis, and not in early injury (Fig. 4 *A* and *B*). These clusters, therefore, likely represent fibroblast states more closely associated with pathology and fibrosis specifically, rather than resolution and healing.

Cluster 6 shares substantial commonality with *Kera^hi^ Fmod^hi^* cluster 7, and expresses highest levels of *Cthrc1* among all the DAFs, an emerging hallmark of pathogenic fibroblasts in fibrosis (15). However, it is unique among the DAFs for its expression of osteopontin (*Spp1*) (Fig. 4*F*), noteworthy as levels of osteopontin correlate with severity in nonocular fibrotic disease (16, 17), it is also the only DAF expressing *Mmp3*. MMP3 can process a number of pro-MMPs and is the first step in MMP-mediated ECM degradation, however as cluster 6 is also the only fibroblast state to express the full combination of protease inhibitors *Timp1*, *Timp2,* and *Timp3* (Fig. 3*D*), *Cthrc1^hi^Mmp3^+^Spp1^+^* cluster 6 may represent a proto-typical universal pathogenic fibroblast enabling a chronic nonprogressive environment that both promotes and destabilizes ECM.

*Fap^+^Fgl2^+^* Cluster 4 represents a fibroblast state with broad functionality, with some immunoregulatory capacity, with high *Csf1 and Fgl2* expression, and some fibrillogenic capacity, with high *Pcolce* and *Ltbp4* expression (Fig. 4*F*). *Ly6c1*, expressed by cluster 2 and cluster 4 DAFs is usually expressed by, and used as a marker to discriminate inflammatory monocytes from homeostatic monocytes. Using flow cytometry, we found that surface expression of Ly6C on fibroblasts isolated from RPE/choroid tissue was increased in fibrosis compared to healthy tissue, and furthermore there was a significant increase in Ly6C^+^ fibroblasts in fibrosis when compared with early injury (Fig. 4*H*). Despite expression of *ly6c1* in cluster 2, there was no significant change in the percentage of Ly6C^+^ fibroblasts present during early stages of disease when compared to healthy tissue. This identifies Ly6C surface expression as a differentiator of fibrosis-associated fibroblasts in subretinal fibrosis. This is even more striking when it is compared to THY1 labeling of the same tissue. *Thy1* is highly expressed in cluster 2 fibroblasts (Fig. 4*H*), however, while there is a significant increase in the percentage of THY1^+^ fibroblasts in both early injury and fibrosis compared to healthy tissue, supporting the observed infiltration of this cluster to sites of injury, there was no significant change in the percentage of THY1^+^ fibroblasts in fibrotic tissue, compared with early injury (Fig. 4*I*). This differential expression of surface Ly6C and THY1 in fibrosis vs in early injury further emphasizes the likely contribution *Fap^+^Fgl2^+^* cluster 4 contributes to subretinal fibrotic processes.

### Periostin Is a Distinguishing Biomarker that Differentiates Subretinal Fibrosis from Wound Repair Across-Species.

To further characterize the *Fap^+^Fgl2^+^* cluster 4, we examined the top DEGs within this cluster (*SI* *Appendix,* Fig. S5*A*). Notably, these top DEGs included a substantial number of genes associated with the matrisome, encompassing various glycoproteins, proteoglycans and collagens. Furthermore, an enrichment of genes encoding ECM regulators, particularly members of the Serpin and ADAM families was noted.

**Fig. 5.**
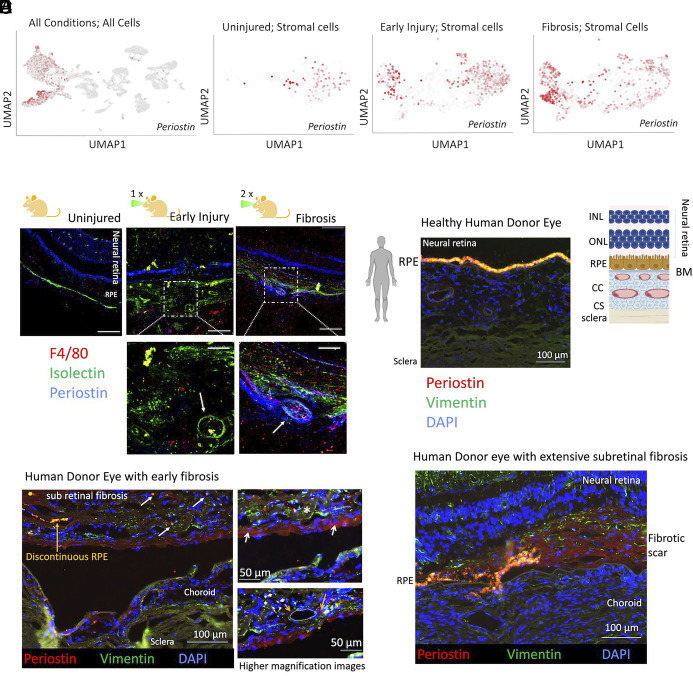
Periostin is a distinguishing biomarker for subretinal fibrosis across mouse and human disease (*A*) UMAP of periostin in all cells and (*B*) stromal cells. (*C*) Retinal cryosections from uninjured (healthy), early injury and fibrotic models stained with isolectin, periostin and F4/80, magnified images shown underneath. (*D*) Retinal cryosections from healthy human donor tissue stained with periostin, vimentin and DAPI. (*E*–*F*) Retinal cryosections from human donor eyes with subretinal fibrosis secondary to nAMD stained with periostin, vimentin and DAPI with a magnified images shown on the right. BM; Bruchs membrane, RPE; retinal pigment epithelium.

One of the ECM genes most highly differentially expressed by the *Fap^+^Fgl2^+^* cluster 4, emerging strongly in fibrosis and mapping with *Ly6c1* expression and is periostin, a protein involved in collagen crosslinking associated with progression of fibrosis in nonocular disease (18) (Fig. 5 *A* and *B*). Immunolabeling of murine tissue cryosections revealed no periostin in healthy uninjured RPE/choroid, or in early injury, whereas we observed strong periostin^+^ labelling across the lesion site in the subretinal fibrosis model, particularly around vessel sites (Fig. 5*C*, blue stain and magnified image). Having thus identified the periostin matrix-generating activity of *Fap^+^Fgl2^+^* fibroblasts, a key pathogenic fibroblast phenotype, in a mouse model of subretinal fibrosis, we wished to examine human subretinal tissue sections for the presence of this pathogenic fibroblast state. Tissue sections of human donor AMD eyes were screened for areas of subretinal fibrosis and compared with healthy human donor eyes. Immunolabeling of human tissue sections similarly revealed no periostin labeling in healthy RPE/choroid (Fig. 5*D*). This was in stark contrast to the apparent increasing gradient of periostin^+^ labeling across the lesion site in subretinal fibrosis secondary to nAMD in line with advancing fibrotic disease (Fig. 5 *E* and *F* and *SI* *Appendix,* Fig. S5*B*). In one human donor eye with CNV and early evidence of fibrosis we observed cells that were double labeled for vimentin and periostin (Fig. 5 *E*, *Left* panel, closed white arrows). We also observed areas of periostin^+^ labelling in fibrous tissue (Fig. 5*E*, open white arrows) alongside fibrous tissue areas that remain free of periostin labeling (Fig. 5 *E*, *Top Right* panel, highlighted with asterisk). We further observed early evidence of periostin at the perimeter of a vessel (Fig. 5 *E*, *Bottom Right* panel, yellow arrows), analogous to that observed in mouse tissue. A second human donor eye with type 2 CNV with subretinal fibrosis and a third with extensive subretinal fibrosis secondary to wet AMD exhibit expansive areas of periostin^+^ fibrous tissue with fibroblastic foci (Fig. 5*F* and *SI* *Appendix,* Fig. S5*B*). Secondary analysis of scRNA-seq of RPE/choroid tissue isolated from aged donor eyes from healthy and nonfibrotic AMD donor eyes (19) demonstrate no periostin expression in stromal cells of nonfibrotic AMD donor eyes, providing further supporting evidence for periostin as a specific biomarker of pathogenic fibroblasts in fibrotic AMD disease (Fig. 5*F* and *SI* *Appendix,* Fig. S6). These data indicate the likely presence of the *Fap4^+^Fgl2^+^* pathogenic fibroblast cluster in human subretinal fibrosis.

## Discussion

Our scRNA seq results provide a systematic atlas of the molecular characteristics of collagen-producing cells in subretinal neovascular lesions, in early injury and fibrotic conditions. Furthermore, *Col1a1*-YFP mice combined with the Micron IV device allow us to noninvasively visualize collagen production in real-time in a longitudinal setting and in conjunction with SHG microscopy, demonstrates that stable collagen fibril and scar formation at retinal neovascular lesions coincides with sustained presence of collagen-producing cells. Transient presence of collagen-producing cells does not produce mature collagen fibers. FACS analysis provides the anatomic, spatial location of the cells that produce high levels of COL1A1, demonstrating that cells resident or migrating through the neural retina produce negligible levels of COL1A1 under conditions of subretinal fibrosis, whereas cells producing high levels of COL1A1 are found in the RPE/choroid tissue. Broadly speaking, endothelial and immune cell populations were found not to express *Col1a1*, while pericytes and fibrocytes produce little to no COL1A1 protein in our models despite expressing *Col1a1* at the mRNA level.

Together, our comparative injury method allowed us the opportunity to investigate subretinal neovascular disease at both an early stage of disease where repair processes are in play and in late stage disease where fibrotic processes are underway. By comparing scRNA seq results between uninjured, repairing, and fibrotic tissue, we were able to identify two umbrella populations of fibroblasts, homeostatic fibroblasts, and disease-associated fibroblasts. HFs were found in all tissue samples while the second population, we term DAFs, appeared only in injured tissue. These DAFs expressed high levels of *Col1a1*, and were marked by high expression of *Pi16*, matricellular components *Fn1*, *Aspn,* and *Ogn* alongside multiple other collagens.

Analysis of fibroblast heterogeneity and identification of specific subpopulations of fibroblasts critical to progression of fibrosis has garnered significant interest over recent years across a variety of organs and diseases (5, 20–22). Subclustering the stromal cells in our scRNA seq dataset revealed twelve phenotypically distinct cell populations, of which four were distinct DAF populations; cluster 2 (*Pi16^hi^ Thy1^hi^*), cluster 4 (*Fap^+^ Fgl2^+^*), cluster 6 (*Cthrc1^hi^Mmp3^+^Spp1^+^*), and cluster 7 (*Kera^hi^ Fmod^hi^*). By comparing the molecular characteristics of HFs to DAFs, we found that ECM-producing and ECM-degrading pathways coexist in DAF clusters in subretinal fibrosis. We verified this parallel expression with confocal microscopy in flatmounts of subretinal fibrotic tissue, labeling for two of the top DEGs in DAFs vs HFs, matrix forming asporin (23) and matrix degrading ADAMTS5. A gradient of fibroblast subsets has been proposed for nonocular fibrotic disease, with ECM overproducing pro-fibrotic fibroblasts at one end and pro-inflammatory fibroblasts at the other (24). Despite each of the DAF clusters expressing genes that would enable parallel ECM-production and degradation, we also found evidence for a polarizing gradient in fibroblast function, with a distinct and nonoverlapping immunoregulatory signature for cluster 2 (*Pi16^hi^ Thy1^hi^*) and a distinct fibrillogenic signature for cluster 7 (*Kera^hi^ Fmod^hi^*) implying these fibroblast clusters represent broadly the pro-inflammatory and pro-fibrotic fibroblasts described previously.

Importantly, our comparative model system uniquely allowed us to identify specific pathogenic characteristics of fibrosis-promoting fibroblasts. All four collagen-producing DAF clusters were found in both injury models, however, terminal state and fate probability mapping strongly suggested that clusters 2 (*Pi16^hi^ Thy1^hi^*) and 7 (*Kera^hi^ Fmod^hi^*) were the main fibroblast fates in early injury indicating these clusters likely represent fibroblasts with repair/healing functions in a regulated environment. Further supporting this interpretation is the parallel expression in cluster 2 of *Pi16*, *Cd55* and *Ackr3*, markers of pan-tissue inflammatory fibroblasts found across all disease models analyzed to date (25).

In direct contrast, fibroblast clusters 4 and 6 are uniquely terminally differentiated in fibrotic tissue, and fate probability mapping indicates these fibroblast clusters expand substantially during fibrosis. Clusters 4 (*Fap^+^Fgl2^+^*) and 6 (*Cthrc1^hi^Mmp3^+^Spp1^+^*) appear most similar to each other among the DAF populations in terms of having greater overlapping gene expression, indicating a broader functionality and less specialization. They appear to hold some immunoregulatory capacity in parallel with some fibrillogenic capacity. Recently, Tsukui et al. reported *Cthrc1* with *Spp1* as a marker of pathologic fibroblasts in pulmonary fibrosis while others have found that CTHRC1^+^ fibroblasts and SPP1^+^ macrophages act synergistically resulting in immuno-regulatory, pro-fibrotic, and EMT mechanisms (7, 26–28). We find *Cthrc1* and *Spp1* most highly expressed in cluster 6 of our DAFs, supporting our interpretation that cluster 6 is a pathologic fibroblast population involved in the process of subretinal fibrosis, and also indicating this is a universal activated fibroblast subset.

Our comparative injury method highlights cluster 4 as a pathological fibroblast cluster in subretinal fibrosis. Cluster composition analysis indicated that the greatest expansion of collagen-producing DAFs in fibrosis was cluster 4, and we confirmed this by flow cytometry. Fibroblast cluster 4 is marked by high expression of ECM components *Postn*, *Ltbp4,* and *Fgl2*, and the highest *Fap* expression across all DAFs. Cluster 4 also has the highest level of *Col6* chain expression across all cells, notable as elevated fragments of COLVI chains, especially endotrophin from *col6a3* are associated with progressive fibrosis in nonocular disease. Periostin is a matricellular protein that has previously been associated with fibrosis across a variety of diseases. Increased periostin expression has in fact been reported in the fibrovascular membrane formed in the inner retina (29), in late stage diabetic disease and secondary to retinal detachment (30). There is a scarcity of data about periostin in subretinal fibrosis, however, Nakama et al. report that periostin is expressed by the RPE in a mouse model of wet AMD and that knockdown of periostin reduces the area of collagen1 staining at the neovascular lesion (31, 32). We replicated Nakama’s finding that periostin is expressed in the RPE early in the response to laser injury in our resolving model. However, we detected no periostin deposition in or around the neovascular lesion itself in our early injury model. In contrast, we found strong periostin labeling in the neovascular lesion in our model of subretinal fibrosis. A major finding of this study is the identification of fibroblast-derived periostin in the neovascular lesion as a distinguishing biomarker unique to subretinal fibrosis as opposed to subretinal injury.

We verified the presence of periostin in human donor eye tissue sections from donors with subretinal fibrosis secondary to neovascular AMD. There was clear periostin deposition at the fibrovascular lesion in the choroid with no periostin detected in healthy donor eye tissue and an apparent gradient of periostin labeling in neovascular lesions with severity of fibrovascular scarring. Periostin is a multifunctional protein (33), but one of its main functions is to enhance collagen crosslinking and fibrillogenesis. FAP also plays a role in remodeling the ECM through its endopeptidase activity creating a more organized and mature collagen and fibronectin matrix (34, 35). Together, it is possible that high expression of FAP and periostin alongside excessive expression of collagens and fibronectin, drive maturation of ECM fiber formation establishing its permanency.

In addition to the collagen-producing fibroblast populations, our methods also identified a cluster of IFN-responsive fibroblasts (cluster 9) that appeared only in the fibrotic model. While these IFN-responsive fibroblasts do appear to be unique to the fibrotic model, the cell cluster is small proportionally to other fibroblast clusters and it remains possible that IFN-responsive fibroblasts are present in early injury model but are too rare to detect. The appearance of the IFN-responsive fibroblast cluster in the fibrotic model is consistent however with the existing literature on fibroblasts in the CNS, where it has previously been shown that fibroblast cell-specific deletion of *Ifngr1* resulted in reduced fibrotic scarring in EAE, a mouse model of multiple sclerosis (36). We did not pursue this IFN-responsive fibroblast cluster as they do not appear to be collagen-producing, they warrant further study.

Our study has many strengths, however, our method of isolating cells for scRNA seq did not favor the collection of RPE cells which is a limitation of this study. In an effort to address the role of RPE in collagen production, we analyzed flatmounted fibrovascular lesions by confocal microscopy. We found RPE differentiation in response to injury, taking on an αSMA^+^ myofibroblast phenotype, however, few αSMA^+^ RPE cells colocalize with areas of Type I collagen. In nonocular fibrotic tissues, cells that produce excess quantities of ECM are thought to arise in response to local injury and migrate to the injured area forming aggregates of fibroblastic foci that drive fibrotic processes (7). We also see evidence of fibroblastic foci in our flatmounts and cryosections. As such, our data imply RPE cells are unlikely to play a major role in the excessive production of collagen. Our finding that fibroblasts are the dominant source of ECM in subretinal fibrosis is a significant advance for the field as this opens more avenues for therapies and extends the focus from RPE mesenchymal transitioning to fibrosis-relevant fibroblasts.

In summary, we have established a collagen-producing cell atlas for subretinal fibrosis. We have identified pathogenic pro-fibrotic fibroblast phenotypes that expand specifically in conditions favoring fibrosis. We show that the molecular characteristics of the fibroblasts from our mouse model are both universal and tissue specific. We identify periostin as a marker capable of discerning between subretinal injury and subretinal fibrosis in mice and humans. This highlights both the translational potential of our resolving and fibrotic mouse models for therapeutic target identification, and implies the pathogenic fibroblast phenotypes we describe for subretinal fibrosis could similarly have pathogenic pro-fibrotic functionality in a broader range of fibrotic conditions.

## Materials and Methods

### Clinical Imaging.

A participant with a confirmed diagnosis of wet AMD and a control participant with no known ocular history were recruited from Progressive Vision Eye Clinic, Dublin, Ireland. Exclusion criteria included prior diagnosis or treatment of any eye disease other than AMD. Macula-centered OCT scans were obtained using the Cirrus HD-OCT 5000 (Carl Zeiss Meditec). Ethical approval was obtained from FHS REC in Trinity College Dublin, Ireland.

### Animals.

All studies were carried out in the Smurfit Institute of Genetics in TCD and adhere to the principles laid out by the internal ethics committee at TCD, and all relevant national licenses were obtained before commencement of all studies. C57Bl/6J mice and *Col1a1*-YFP mice (B6.Cg-Tg(Col1a1*3.6-Topaz)2Rowe/J, Strain 017466) mice were sourced from Jackson Laboratory and bred on-site. *Col1a1*-YFP mice were kept as a hemizygous line.

### Laser-Induced Choroidal Neovascularization (LCNV) Model.

Mouse pupils were dilated with 1% tropicamide and 2.5% phenylephrine and anesthetized with ketamine/medetomidine (100/0.25 mg/kg). LCNV was carried out using the Micron IV platform (532 nm, 300 mW, 100 ms, 50 μm spot size, 3 to 4 spots per eye) in 8 to 12 wk old *Col1a1*-YFP mice. In mice receiving the two stage LCNV model, 7 d after the initial LCNV, mice received a second set of laser burns (532 nm, 250 mW, 100 ms, 50 μm spot size, 3 to 4 spots per eye) directly over the initial lesions.

### In Vivo YFP Imaging.

Pupils from *Col1a1*-YFP mice were dilated with 1% tropicamide and 2.5% phenylephrine and anesthetized with ketamine/medetomidine (100/0.25 mg/kg). YFP imaging was performed using the Micron IV platform using the YFP filter.

### Tissue preparation for scRNAseq analysis and flow cytometry.

Mouse eyes were collected in 5% fetal bovine serum (FBS) in PBS and dissected to remove the cornea and lens. Retina and RPE/choroid were digested for 40 min at 37 °C, filtered through a 70 um strainer, centrifuged for 5 min at 1,000 rpm and resuspended in 0.04% bovine serum albumin (BSA) in PBS. For scRNA seq analysis, 8 eyes were pooled and cells were stained with PI and DRAQ5 and gated for PI negative, DRAQ5 positive, and YFP positive/YFP negative cells and sorted on the BD FACSAria Fusion flow cytometer. For flow cytometry, 2 eyes were pooled per sample with n = 3 samples. Cells were blocked in mouse Fc block for 10 min, and stained with Live/Dead Aqua (1:1,000, Life Technologies) for 15 min. Following 1% FBS in PBS washes, cells were incubated in fluorochrome-labeled primary antibodies diluted 1:50 in 1% FBS in PBS for 20 min at 4 °C. Cells were washed in 1% FBS in PBS and flow cytometry was carried out on a BC LSR Fortessa cell analyzer and analyzed using FlowJo software.

### Immunohistochemistry of Human Tissue.

Human donor eye tissue was obtained by the Iowa Lions Eye Bank (Iowa City, IA) with full consent from the next of kin. All experiments were performed in compliance with the Declaration of Helsinki. Immunohistochemistry was performed on paraformaldehyde-fixed frozen macula tissue sections from three AMD donors and three age-matched controls. Tissue sections were visualized on an Olympus BX41 microscope.

### Secondary Harmonic Generation Imaging.

Multimodal multiphoton imaging was performed using a custom-built upright laser scanning microscope as previously described (37). SHG and two-photon excited fluorescence (2PEF) signals were detected simultaneously in the forward direction by photon-counting photomultiplier tubes (P25PC, Sentech, UK). Images were acquired using 15 mW excitation power, 5 µs pixel dwell time and 420 nm pixel size.

### scRNA Seq.

scRNA seq was performed using the 10X Genomics Chromium Single Cell 3′ v3 platform. YFP^+^ and YFP^−^ cells sorted from RPE/choroid tissue were resuspended in 0.04% BSA in PBS and loaded onto the Chromium controller. Chromium Single Cell 3′ v3 reagents were used for library preparation according to the manufacturer’s protocol. Sequencing data were processed using Cellranger and mapped to the mouse genome (mm10). Secondary analysis utilized *Scanpy* (v1.9.6). Raw counts were normalized by total cell count and log1p-transformed, with 4,000 highly variable genes selected for downstream analysis. Initial Leiden clustering (resolution 1) identified cell populations, which were annotated using known markers. Stromal cells were further subclustered at various Leiden resolutions (0.05 to 1); a resolution of 0.5 was chosen based on Silhouette scores and manual inspection. GSEA was performed via gseapy (GO_Biological_Process_2021) and GSEA Preranked software. Fate probabilities and terminal states were mapped using Palantir (cellrank2). For comparative analysis, a pan-tissue Fibrosis Atlas was integrated with mouse lung data using scANVI/trVAE (scArches) to mitigate batch effects. Subsequent clustering and dimensionality reduction were performed on the model’s latent space. Visualizations were generated using Tibco Spotfire and Scanpy.

## Supplementary Material

Appendix 01 (PDF)

## Data Availability

Single cell RNA-sequencing data have been deposited in GEO (Accession GSE318755) (38).
