# Supplementary material for: Collagen-producing eye cell atlas reveals distinct fibroblast fates in early injury vs. fibrotic subretinal disease
Source: Proc Natl Acad Sci U S A. 2026 Jun 26;123(26):e2519056123. doi: 10.1073/pnas.2519056123 (PMC13320955; doi:10.1073/pnas.2519056123)
Supplement: Supplementary file 1 — Appendix 01 (PDF) [file pnas.2519056123.sapp.pdf]

Collagen-producing eye cell atlas reveals distinct fibroblast fates in early injury versus fibrotic subretinal disease

Authors: Ema Ozaki[1,2,3], Said Aktas[4], Kelly Mulfaul[5,6], Kiva Brennan[1,2], Christophe Roubeix[3], Sarah Palko[1,2], Katie Robb[1,2,7,8], Tai-Hsien Ou Yang[9] Marie-Claire Schanne-Klein[10], Anna Toidze[3,11], Avril Watson[1,2], Mark Cahill[7,8], Peter D. Westenskow[3], Derrick Feenstra[3*], Sarah L. Doyle[1,2*]

***Joint and corresponding author details:**

Name: Dr. Sarah Doyle
Email sarah.doyle@tcd.ie
Telephone: +353 1 8963011
Address: 4.43 Lloyd Building,
Trinity College Institute of Neuroscience,
School of Medicine,
Trinity College Dublin,
Dublin 2, Ireland.

Name: Dr. Derrick Feenstra
Email: derrick.feenstra@roche.com
Telephone: +41 79 880 09 81
Address: F. Hoffmann-La Roche Ltd.
Grenzacherstrasse 124
CH-4070 Basel
Switzerland

Materials and Methods

Clinical Imaging

A participant with a confirmed diagnosis of wet AMD and a control participant with no known ocular history were recruited from Progressive Vision Eye Clinic, Dublin, Ireland. Exclusion criteria included prior diagnosis or treatment of any eye disease other than AMD. Data obtained from the medical records included the duration of wet AMD diagnosis, history of anti-VEGF injections and details of prior treatment interval. Informed consent was obtained from all participants. Participants underwent visual acuity (VA) and intraocular pressure (IOP) measurements before pupillary dilation with 1% tropicamide drops. Fundus images were acquired with the Optos Silverstone (Optos PLC), capturing an ultrawide 200-degree field of view. Macula-centred OCT scans were obtained using the Cirrus HD-OCT 5000 (Carl Zeiss Meditec). All retinal images and OCT scans were reviewed by a retinal specialist to identify abnormalities. Ethical approval was obtained from FHS REC in Trinity College Dublin, Ireland.

Animals

All studies were carried out in the Smurfit Institute of Genetics in TCD and adhere to the principles laid out by the internal ethics committee at TCD, and all relevant national licenses were obtained before commencement of all studies. C57Bl/6J mice and *Col1a1*-YFP mice (B6.Cg-Tg(Col1a1*3.6-Topaz)2Rowe/J, Strain 017466) mice were sourced from Jackson Laboratory and bred on-site. *Col1a1*-YFP mice were kept as a hemizygous line.

Laser-induced choroidal neovascularization (LCNV) model

Mouse pupils were dilated with 1% tropicamide and 2.5% phenylephrine and anesthetized with ketamine/medetomidine (100/0.25 mg/kg). LCNV was carried out using the Micron IV platform (532 nm, 300 mW, 100 ms, 50 um spot size, 3-4 spots per eye) in 8-12 week old *Col1a1*-YFP mice. In mice receiving the two stage LCNV model, 7 days after the initial LCNV, mice received a second set of laser burns (532 nm, 250 mW, 100 ms, 50 um spot size, 3-4 spots per eye) directly over the initial lesions. Males were used in the scRNA seq analysis, while male and female mice were used for immunohistochemistry and flow cytometry analysis.

In vivo YFP imaging

Pupils from *Col1a1*-YFP mice were dilated with 1% tropicamide and 2.5% phenylephrine and anesthetized with ketamine/medetomidine (100/0.25 mg/kg). YFP imaging was performed using the Micron IV platform using the YFP filter.

Immunohistochemistry

For retinal cryosections, eyes were fixed in 4% paraformaldehyde for 1.5 h at room temperature, followed by three PBS washes. Cornea and lens were removed and the eyecups were cryoprotected in 20% sucrose for 1 h, followed by 30% sucrose overnight at 4°C. Eyes were subsequently embedded and frozen in an optimum cutting temperature compound. 12 µm sections were collected onto Polysine slides using a cryostat. For RPE flatmounts, the cornea, lens and retina were carefully removed, and four incisions were made into the eye cup to flatten out the RPE/choroid tissue. RPE flatmounts were fixed for 30 min in 4% paraformaldehyde, followed by three PBS washes. Cryosections and flatmounts were permeabilized and blocked in 10% normal goat serum (NGS) and 0.1% Triton in PBS for 1 h at room temperature. The samples were then incubated overnight at 4°C with primary antibody diluted in 5% NGS. Primary antibodies used were Collagen Type I (1:200, Rockland Immunochemicals, 600-401-103), αSMA-Cy3 (1:200, Merck, C6198), Vimentin (1:200, RnD systems, MAB2105), LOXL2 (1:100, Abcam, ab96233), Adamts5 (1:100, Abcam, ab41037), Asporin (1:100, Novus Biologicals, NB100-1514) and Periostin (1:200, Abcam, ab14041). For biotin-conjugated Collagen-hybridizing peptides (1:15, 3Helix, BIO60CTL) staining, peptides were first heated to 80°C for 5 mins in 10% NGS, followed by cooling on ice for 90 sec, before immediately incubating on samples. After three PBS washes, the samples were incubated with Alexa Fluor 594- and Alexa-405-conjugated goat anti-rabbit, Alexa Fluor 405-conjugated goat anti-rat and Alexa Fluor 594-conjugated donkey anti-goat secondary antibodies (1:500; Invitrogen) or with Alexa Fluor 594-conjugated streptavidin and Alexa Fluor 488-conjugated Isolectin GS-IB4 (1:500) diluted in 5% NGS for 2 h. The samples were mounted with Hydromount (VWR) mounting medium and analyzed using a confocal microscope (Zeiss LSM 710).

Tissue preparation for scRNAseq analysis and flow cytometry

Flow cytometry was performed on retina and RPE/choroid tissue from *Col1a1*-YFP mice and WT mice 5 days after 1x LCNV, 17 days after 2x LCNV and in uninjured tissue. Mouse eyes were collected in 5% fetal bovine serum (FBS) in PBS and dissected to remove the cornea and lens. The retina and RPE/choroid were collected in digestion buffer (HBSS supplemented with 5% FBS, 10 mM HEPES, 1.5 mg/ml Collagenase and 0.1 mg/ml DNase I). Samples were digested for 40 min at 37°C, passed through a 70 um cell strainer, centrifuged for 5 min at 1,000 rpm and resuspended in 0.04% bovine serum albumin (BSA) in PBS.

For scRNA seq analysis 8 eyes were pooled and cells were stained with PI and DRAQ5 and gated for PI negative, DRAQ5 positive and YFP positive/YFP negative cells and sorted on the BD FACSAria Fusion flow cytometer. For staining with antibodies 2 eyes were pooled per sample with n = 3 samples. Cells were blocked in mouse Fc block for 10 min, and stained with Live/Dead Aqua (1:1000, Life

Technologies) for 15 min. Following 1% FBS in PBS washes, cells were incubated in fluorochrome-labeled primary antibodies diluted 1:50 in 1% FBS in PBS for 20 min at 4°C. Antibodies used were CD90-APC Cy7, Ly6C-PerCP Cy5.5, CD140a-APC and CD146-PE Cy7. Cells were washed in 1% FBS in PBS and flow cytometry was carried out on a BC LSR Fortessa cell analyser and analysed using FlowJo software.

Staining of human tissue

Human donor eye tissue was obtained by the Iowa Lions Eye Bank (Iowa City, Iowa, USA) with full consent from the next of kin. All experiments were performed in compliance with the Declaration of Helsinki.

Immunohistochemistry of human tissue

Immunohistochemistry was performed on paraformaldehyde-fixed frozen macula tissue sections from 3 AMD donors and 3 age-matched controls (**Table 1**) with antibodies directed against Vimentin and Periostin. Tissue sections were blocked in 0.1% bovine serum albumin for 15 minutes and incubated with an anti-mouse Vimentin antibody (1:100, Sigma Aldrich, V2258), and an anti-rabbit Periostin antibody (1:500, Abcam, AB14041-1001) for 1 h at room temperature. Followed by three washes with PBS prior to a 30 minute incubation with secondary antibodies Alexa Fluor 488 Donkey anti-mouse 1:200 (Invitrogen) and Alexa Fluor 546 donkey anti-rabbit 1: 400 (Invitrogen) in PBS with DAPI. Tissue sections were washed three times and mounted with Aquamount. Tissue sections were visualized on an Olympus BX41 microscope.

**Disease Status**	**Age**	**Sex**
Control	79	Male
Control	86	Female
Control	85	Female
CNV/ fibrosis	81	Male
Type 2 CNV with fibrosis	92	Female
Wet AMD extensive fibrosis lesion	82	Female

Table 1: Characteristics of human donors. Three donors with age-related macular degeneration and three age-matched controls. Column 1 describes the stage of AMD, column 2 the age of donor, and column 3 the sex of donor.

Secondary Harmonic Generation imaging

Multimodal multiphoton imaging was performed using a custom-built upright laser scanning microscope as previously described[36]. The circularly polarized laser excitation was set at 860 nm and focused using a 25 x, 1.05 NA water immersion objective lens (XLPLN25XWMP, Olympus), resulting in a lateral resolution of 0.335 µm and an axial resolution of 1.250 µm full width at half maximum (FWHM). Second Harmonic Generation (SHG) and two-photon excited fluorescence (2PEF) signals were detected simultaneously in the forward direction by photon-counting photomultiplier tubes (P25PC, Sentech, UK), using appropriate dichroic mirrors and spectral filters. Images were acquired using 15 mW excitation power, 5 µs pixel dwell time and 420 nm pixel size.

scRNA seq

scRNA seq was performed using the 10X Genomics Chromium Single Cell 3′ v3 platform. YFP^+ and YFP^- cells sorted from RPE/choroid tissue were resuspended in 0.04% BSA in PBS and loaded onto the Chromium controller. Chromium Single Cell 3' v 3 reagents were used for library preparation according to the manufacturer's protocol.

Data Processing

Sequencing data was processed using Cellranger and mapped to the mouse genome (mm10). The secondary analysis starting from raw counts was performed with the *Scanpy* package (version 1.9.6)[37]. Quality control measures included filtering out cells with high mitochondrial gene expression, cells with less than 200 or more than 10,000 expressed genes, and genes expressed in less than 3 cells. The processed data was normalized per total cell counts and log1p-transformed. Highly variable genes (n=4000) were selected for downstream analyses. Principal Component Analysis (PCA), t-Distributed Stochastic Neighbor Embedding (tSNE), and Uniform Manifold Approximation and Projection (UMAP) were employed for dimensionality reduction.

Clustering and cell type annotation

Leiden clustering was performed to identify distinct cell populations within the dataset using default resolution 1. Clusters were annotated by using known marker genes. The subset of stromal cells was re-analyzed and clustered using Leiden clustering at resolutions from 0.05 to 1, and the clustering quality was assessed using the Silhouette score and by manual inspection. A resolution of 0.5 was found to be optimal and therefore used for downstream analysis and visualization.

Identification of cluster-specific genes and gene set enrichment analysis (GSEA)

Cluster-specific genes were identified using the Wilcoxon test. GSEA was performed using the gseapy package with GO_Biological_Process_ 2021 from Gene Ontology as reference, as well as the GSEA Preranked software.

Fibrosis Atlas Construction

A pan-tissue Fibrosis Atlas was constructed using stromal cells from mouse lung (PMID: 32317643)[7] with this study. The scANVI and/or trVAE methods from the *scArches* package[38] were used to integrate the datasets coming from various studies and tissues and remove batch effects. Downstream clustering at various resolutions and dimensionality reduction was performed on the latent space of the model.

Terminal stated and fate probability mapping

Identification of terminal states and fate probability mapping was performed using palantir from the cellrank2 package[39].

Visualization

Plots were generated either with the Tibco Spotfire software (UMAPs, bar charts) or in Python using the Scanpy package (dot plots, radar plot)

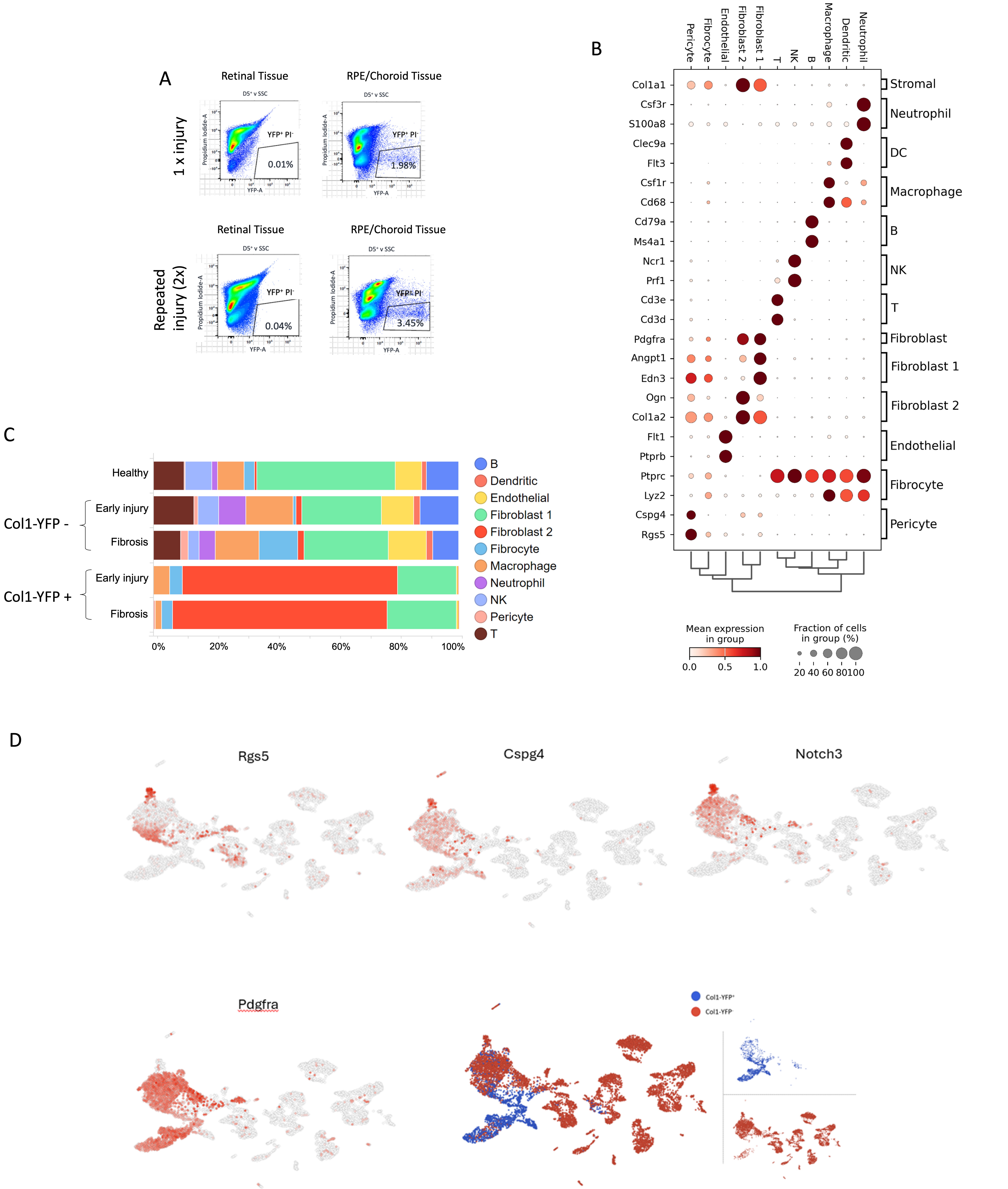

SI Figure 1: (**A**) Quantification of YFP$^+$PI$^-$ cells in retina and RPE/choroid tissue in *Col1a1*-YFP mice 5 days post 1x LCNV induction and 17 days post repeated 2x LCNV induction. (**B**) Dot plot showing expression levels in each cluster of representative marker genes. (**C**) Bar chart displaying percentages of cell type in each sample. (**D**) Uniform manifold approximation and projection (UMAP) plot of all cells showing gene expression levels of Rgs5, Cspg4, Notch3, *Pdgfra* in all cell clusters and UMAP of all cells, in all conditions, colour-coded by YFP$^+$ (blue) and YFP$^-$ (red) samples.

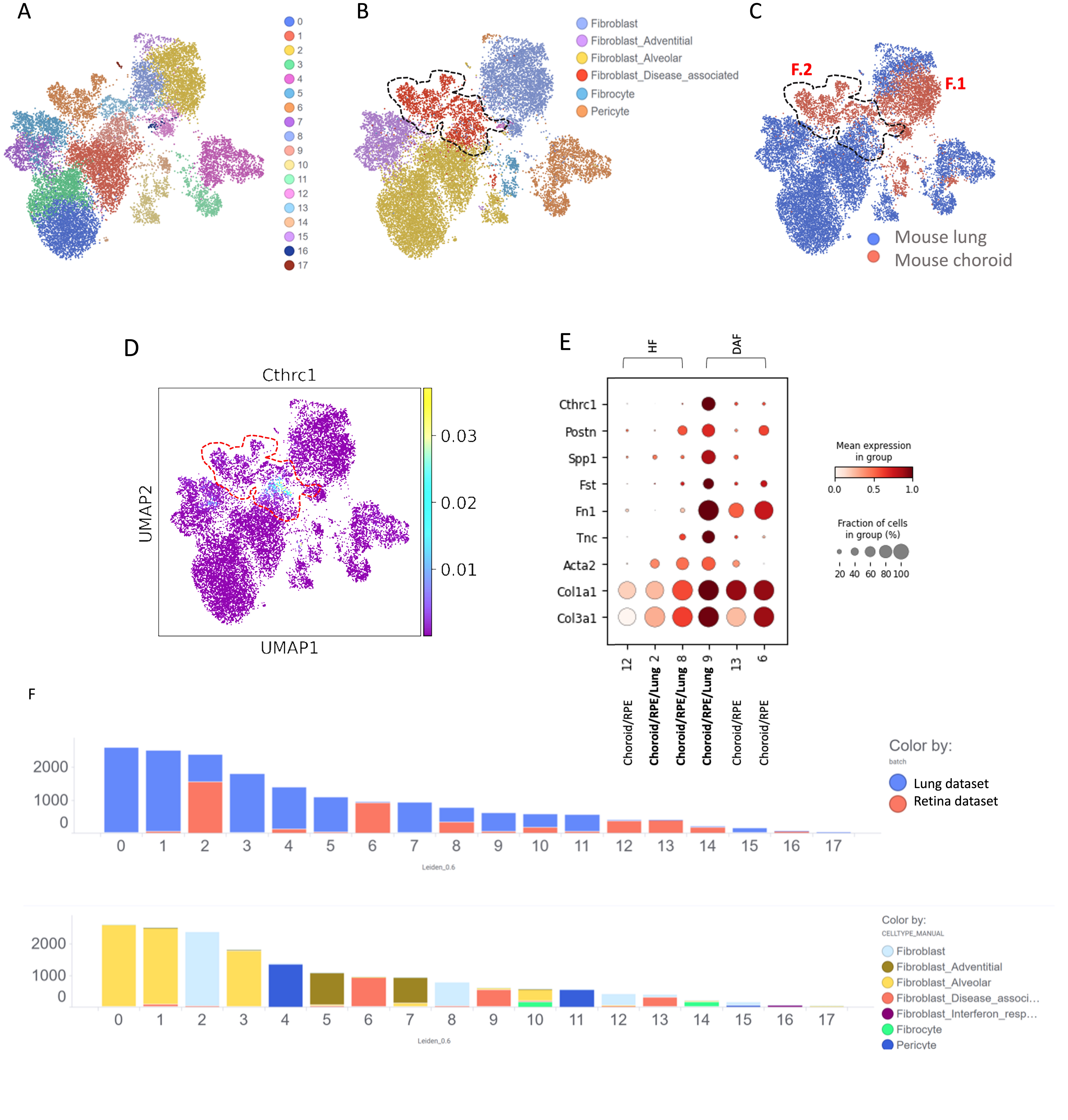

SI Figure 2: Stromal cell cross-organ integration study and reclustering analysis confirms distinct DAF states in subretinal fibrosis. (**A**) UMAP integrating all stromal cells from our study with a lung fibrosis scRNA seq study, colour-coded by the 5 stromal clusters generated in the integration study. (**B, C**) UMAP of integration study colour-coded by (**B**) cell phenotype, (DAF phenotype is outlined in black-dashing) and by (**C**) study source. (**D**) UMAP of integrated study with mean expression of *cthrc1*. DAF cluster is highlighted in red-dashing. (**E**) Dot plot of pathogenic fibroblast marker genes. **(F)** Barcharts of integration of all stromal cells from our study with a lung fibrosis scRNA seq study, colour-coded by study origin or cell phenotype.

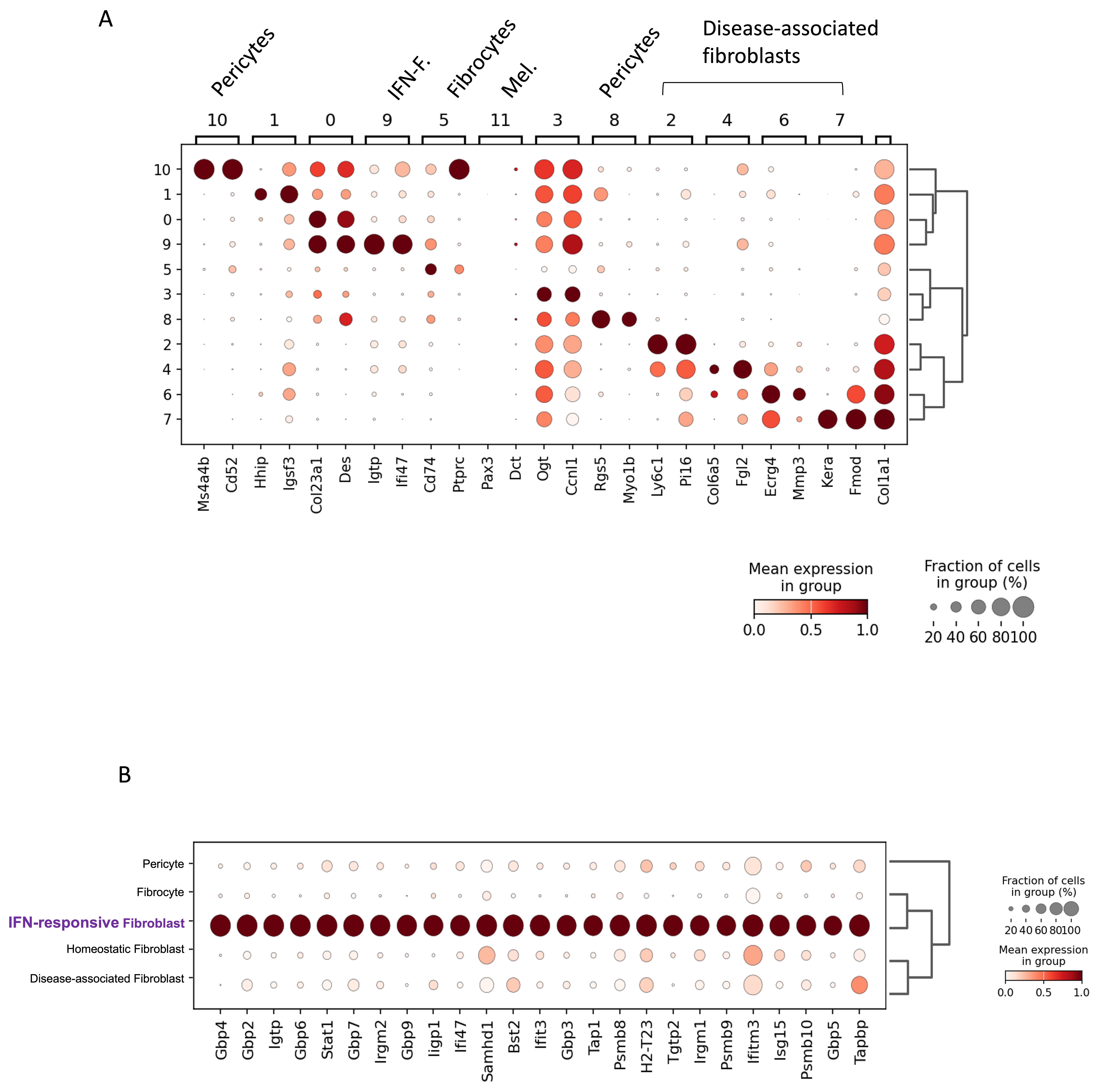

SI Figure 3: Reclustered *Col1a1⁺* cells from the current subretinal-RPE/choroid study produced 12 new clusters

(**A**) dot plot showing expression levels in each cluster of representative marker genes, (**B**) UMAP. Dot plot showing expression levels of top DEGs from cluster 9 in all stromal clusters.

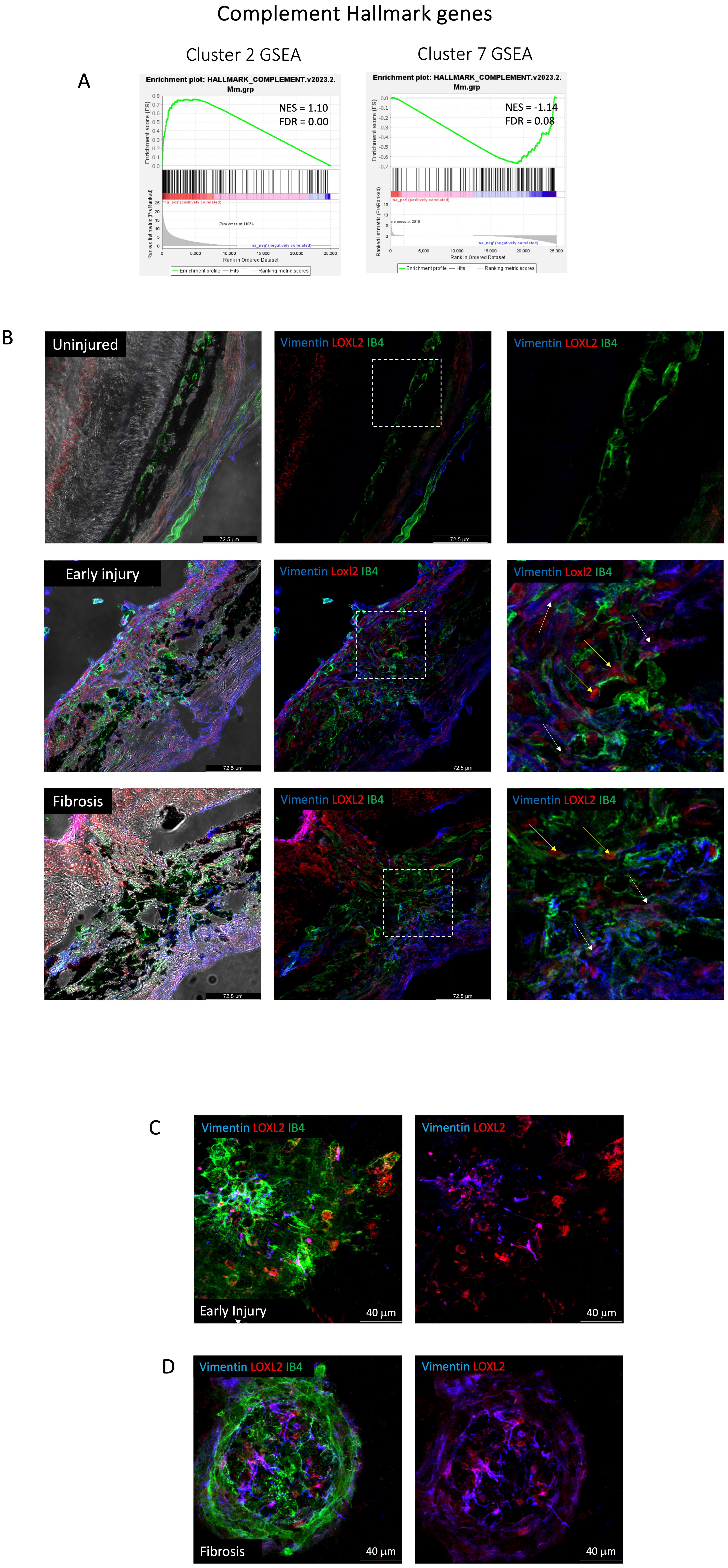

SI Figure 4: (**A**) Gene set enrichment analysis (GSEA) pathway analysis of cluster 2 and cluster 7, generated using the GSEA Preranked software. Normalized expression score and false discovery rate (FDR) are indicated on graphs. (**B**) Retinal cryosections from uninjured (healthy), 5 days (early injury, 1x) and 17 days (fibrosis, 2x) post LCNV WT mice stained with vimentin, LOXL2 and IB4, with magnified images shown on the right. White arrows depict areas of vimentin and LOXL2 co-staining. Yellow arrows depict LOXL2 staining without co-localization to vimentin. (**C, D**) RPE flatmounts from WT mice (**C**) 5 days (early injury, 1x) and (**D**) 17 days (fibrosis, 2x) post LCNV stained with vimentin, LOXL2, IB4. White arrows depict areas of vimentin and LOXL2 co-staining.

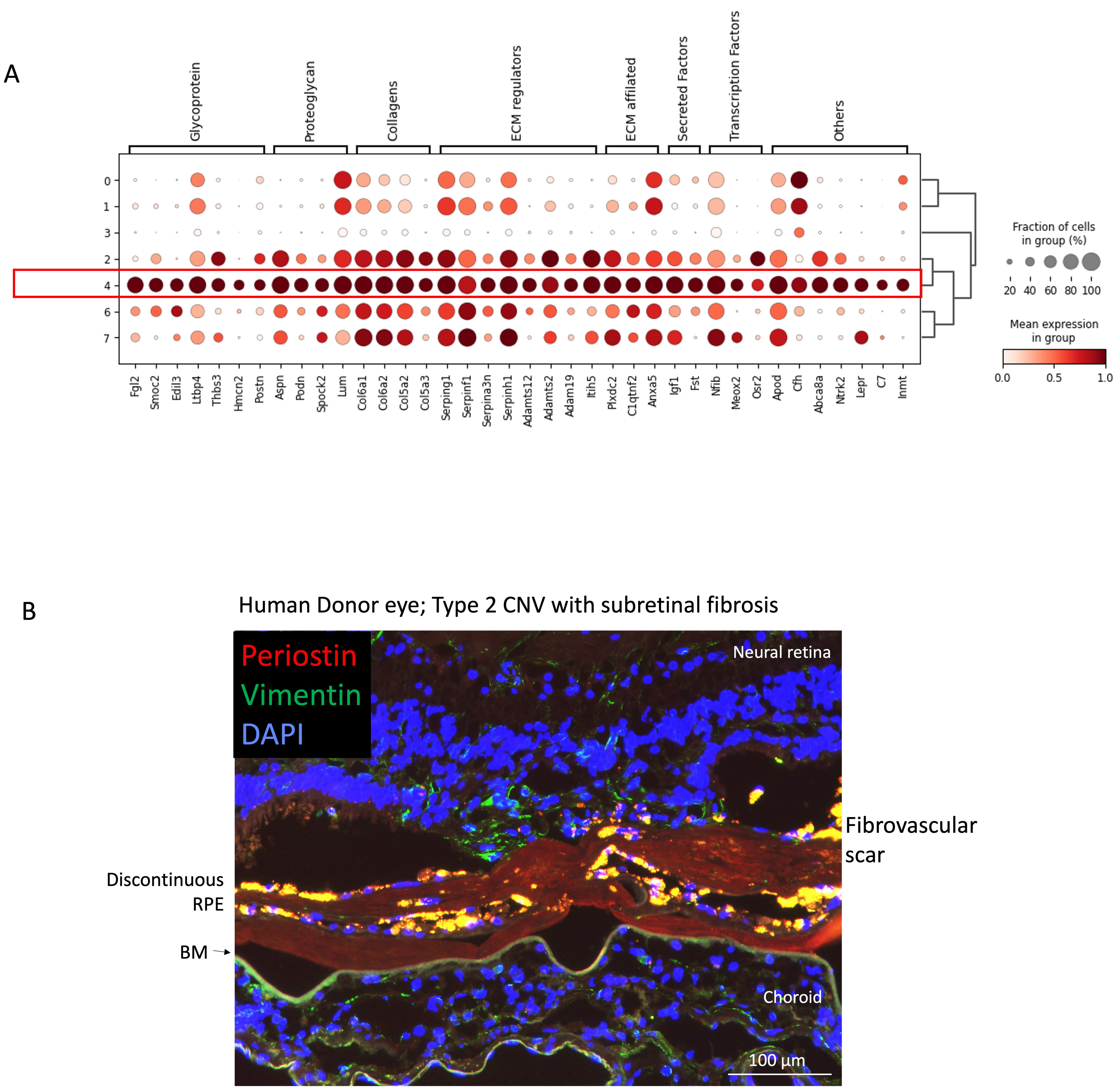

SI Figure 5: (A) Dot plot showing top DEGs in cluster 4 amongst all fibroblast clusters. **(B)** Retinal cryosections from human donor eyes with subretinal fibrosis secondary to nAMD stained with periostin, vimentin and DAPI with a magnified images shown on the right. BM; Bruchs membrane, RPE; retinal pigment epithelium**.**

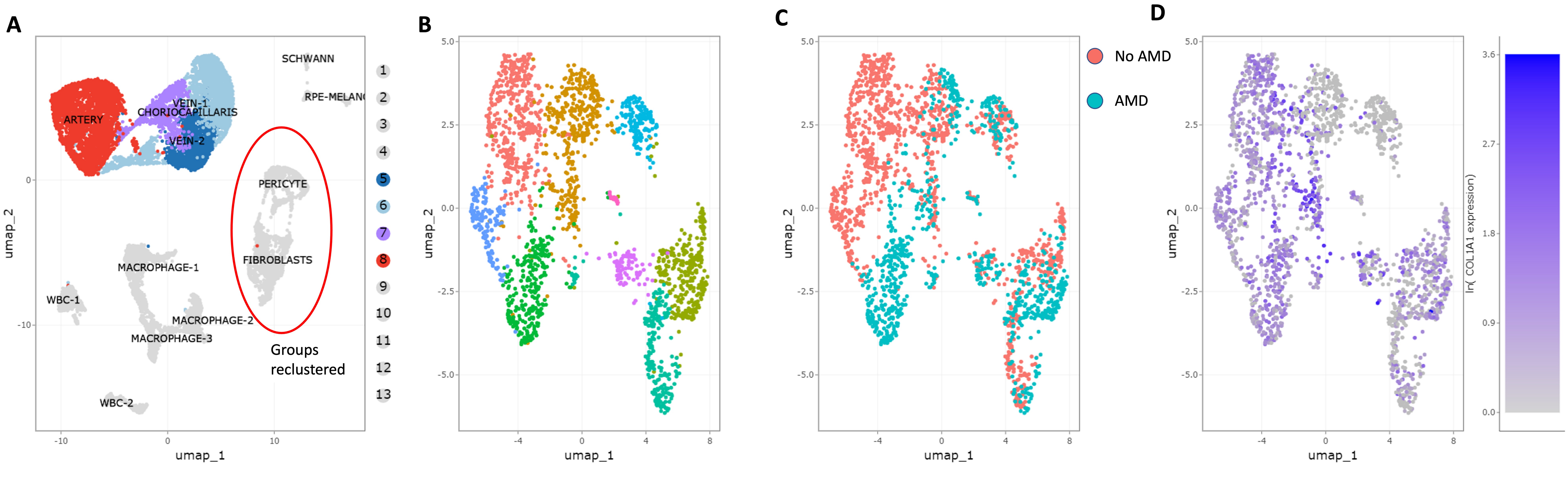

SI Figure 6: (**A**) UMAP from a *Spectacle* database from a human AMD study[40] depicting all cell clusters of the RPE/choroid tissue with the pericyte and fibroblast cluster marked with a red circle. (**B**) UMAP of the reclustered pericyte and fibroblast clusters. (**C**) UMAP of the pericyte and fibroblast clusters colour coded by cells coming from AMD and No AMD samples. (**D**) UMAP of the pericyte and fibroblast clusters showing *Col1a1* expression.
